# A review of feline infectious peritonitis virus infection

**DOI:** 10.14202/vetworld.2024.2417-2432

**Published:** 2024-11-05

**Authors:** Tridiganita Intan Solikhah, Qurrotul Aini Dwi Agustin, Ratmasari Alifina Damaratri, Della Ayuke Fika Siwi, Ghulam Naufal Rafi’uttaqi, Vincent Angelino Hartadi, Gahastanira Permata Solikhah

**Affiliations:** 1 Division of Veterinary Clinic, Department of Health and Life Sciences, Faculty of Health, Medicine, and Life Sciences, Universitas Airlangga, Banyuwangi, Indonesia; 2 Cahaya Pet Clinic, Veterinarian, Mojokerto, Indonesia

**Keywords:** clinical, feline coronavirus, feline infectious peritonitis virus, infectious disease

## Abstract

Feline infectious peritonitis (FIP) is an infectious disease characterized by non-specific laboratory changes and clinical signs. Clinical symptoms include anorexia, jaundice, fever, and weight loss. Moreover, some lesions are found in the digestive and respiratory systems. FIP, whose virulence varies, cannot be distinguished using several diagnostic methods. Moreover, feline coronaviruses (FCoVs) can be classified into two serotypes based on differences in their amino acid sequences, spike (S) protein sequences, and antibody (Ab) neutralization. There are two pathotypes, namely those caused by FCoV, which are often referred to as feline enteric coronavirus and FIP virus (FIPV). Furthermore, FIPV infection can be caused by sub-neutralizing levels of anti-FIPV S Abs. Therefore, a supporting diagnosis is needed to confirm FIP because there are no specific symptoms. This review aimed to provide updated information on FIP, including epizootiology, clinical and pathological characteristics, pathogenesis, hematology, clinicopathological and imaging features, pathological features, experimental infection, treatment and prevention, infection and immunity, animal and public health considerations.

## Introduction

Based on histological abnormalities, infectious peritonitis in cats was initially identified as a distinct disease in 1963 [1]. It was not until 1970 that the exact cause of this illness was identified, as viral particles were found in 12 of 25 cats with feline infectious peritonitis (FIP) [2]. The components included viruses that induce tropicalis in macrophages, virions housed within vesicles and cisterns, and the Golgi apparatus. Notably, there is an absence of viral plasma membrane budding and elongated cylindrical protrusions extending outward from the viral particle. FIP is classified within the *Coronaviridae* family. The etiologies of both diseases might be linked to coronaviruses and retroviruses, including mouse hepatitis virus (MHV) and feline leukemia virus (FeLV) (FIP virus [FIPV]), as suggested by the structural similarities between MHV and suspected FIPV, disease parallels between FIP- and MHV-related conditions, and potential connections between the two. The confirmation of the viral origin and elucidation of the pathogenesis of FIP took several years because of difficulties in isolating FIPV from clinical cases and cultivating the virus *in vitro*. Initially, peritoneal exudate from cell cultures was employed to grow viruses *in vitro*, which subsequently proliferated in small intestine cultures of cats. In 1979, the development and progression of a virus-causing experimental FIP inoculation in cats were observed in a continuous cell line of feline origin. This virus is also referred to as coronavirus [3]. Approximately 0.3%–1.4% of cat deaths in veterinary institutions are due to FIP [3–5]. FIP can be difficult to diagnose due to the absence of pathognomonic clinical signs or laboratory changes, especially if there is no effusion. However, given that the disease is fatal if left untreated, obtaining an accurate diagnosis is critical [1].

The significance of exploring FIP serves as a foundational reference, enabling readers to conduct research on FIP, thereby enhancing their understanding of its various aspects. Specifically, this includes the pathogenesis theory of FIPV infection, causative agents, epizootiology, clinical manifestations, hematology, clinicopathological and pathological features, experimental research, treatment prevention, and FIP immunity. This review aims to provide detailed explanations concerning the pathogenesis of FIPV infection, causative agents, epizootiology, clinical manifestations, hematology, clinicopathology, experimental pathological features, treatment and prevention strategies, FIP infection, and immunity based on diverse studies.

Feline enteric coronavirus (FECV) and FIPV were initially thought to be distinct viral species. Subsequent studies have suggested that FECV and FIPV are closely related viruses with different virulence traits. When FIPV FECV isolates from the same cattery were compared with feline coronavirus (FCoV) sequences from different caterers/geographical regions, sequence analyses of the two types exhibited significant resemblance [4]. These findings support the theory that FIPV evolved from FECV through specific mutations in the viral genome of infected individual cats [5]. Animal tests further substantiate the “internal mutation” theory [6]. FIP represents a small proportion of F-CoV-infected cats that develop severe disease characterized by vasculitis within a syndrome of serositis and pyogranulomatous inflammation [7].

This review aimed to provide updated information on FIP, including epizootiology, clinical and pathological characteristics, pathogenesis, hematology, clinicopathological and imaging features, pathological features, experimental infection, treatment and prevention, infection and immunity, animal and public health considerations.

## Causative Agent

The virulent forms or biotypes of FECV and FCoV are commonly referred to as FIPV. Ferret systemic coronavirus is responsible for a disease similar to FIP, whereas ECE causes epizootic catarrhal enteritis [8]. Although the exact cause of FIPV remains unknown, mutations in the FECV genome that leads to amino acid alterations in the encoded proteins are believed to play a significant role [9]. The term FCoV has been used broadly for all distinct FCoV classifications based on antigenic properties and biological varieties [10]. Under this classification, FCoV is divided into two biotypes: FIPV and FECV [11]. FIPV shares similarities with feline retroviruses that cause acute infectious sarcomas. A mutant form of this virus has been detected exclusively in tumors and is not transmitted horizontally in nature, unlike primary FeLV, which is shed in various bodily excretions and secretions, facilitating horizontal transmission. FIPV exhibits strong attachment to cells and tissues, making fecal or urine discharge possible only under rare circumstances [12].

The genetic compositions of FCoVs share similarities with the genetic materials. The average RNA strand comprises approximately 29,000 nucleotide units. The dataset includes 11 potential open reading frames (ORFs) or genes. These consist of replicated non-structural components featuring two major ORFs: Four structural ORFs responsible for coding spike (S), coat, membrane, and nucleocapsid proteins and five accessory ORFs identified as 3a-c and 7a,b. Notably, the presence of Gen 7a does not appear crucial for virulence, as evidenced by the observations of FECV and FIPV field strains lacking the functional 7a gene [13]. The role of the mutated 7b gene differs, but minor deletion mutations in 7b gene were identified in 8 of 32 associated isolates related to enteric, infectious, and FIP conditions [14].

FIPV is categorized into two serotypes, type I and II, depending on the antibodies (Abs) that neutralize the virus: Type I and type II [15]. Serotype I FIPV exhibits a unique S protein specific to cats, whereas serotype II is a combination of cat and dog enteric coronaviruses [14]. Globally, serotype I FIPV is more prevalent; however, in Japan, type II viruses comprise >30 of 100 isolates. Furthermore, type II variants appear more prevalent and readily adaptable to tissue culture, whereas type I variants have a higher tendency to induce clinical FIP [16].

## Epizootiology

FIP was clinically recognized before 1962 when Jean Holzworth first described its clinical presentation and identified characteristic lesions under the term “Chronic fibrinous peritonitis.” The first comprehensive study on this condition was carried out in 1966. Subsequently, FIP has been reported globally across five continents: North America (the United States and Canada), Africa (South Africa and Senegal), Asia (Japan), Oceania (Australia), and Europe (Great Britain, Ireland, the Netherlands, Germany, Belgium, Switzerland, and France) [17].

FIP commonly affects domestic cats younger than 2 years [18]. The incidence was equal for both male and female cats. Domestic breeds are more sensitive to FIP than other breeds. The disease affects all members of the Felidae family [17]. Similar cases have been observed in various feline species, including the African lion, mountain lion, leopard, cheetah, jaguar, lynx, serval, caracal, European wild cat, sand cat, and Pallas cat [19].

## Clinical and Pathological Characteristics of FIP

Moyadee *et al*. [18] identified two primary forms of FIP: Effusive and non-effusive. The findings of this study highlighted the prevalent clinical features of cats with FIP, which included abdominal distension (68%), depression (60%), dehydration (58%), anorexia (53%), and dyspnea (42%). Effusive FIP is characterized by fluid accumulation in the abdomen, pleura, and/or other body cavities, such as the pericardial cavity, renal subcapsular space, scrotum, and heart due to increased vascular permeability caused by blood vessel inflammation. Cats may exhibit symptoms such as dyspnea and abdominal distension. These clinical features stem from the vascular and perivascular alterations provoked by the virus, which manifest in two primary evolutionary forms: “Wet” or exudative and “dry,” which lacks fluid accumulation in cavities. The wet form involves general alterations (fever, apathy, and weakness), dyspnea, exudative peritonitis (enlarged abdominal volume, positive abdominal balloon test, inflammatory fluid on peritoneal puncture with total protein exceeding 3 g/dL), and high mortality. Conversely, the dry form represents chronic progression with atypical symptoms that vary based on primary location. In cases with nerve involvement, functional neurological signs are predominant, notably behavioral changes (apathy) and motility issues (otolaryngological weakness, paresis, paralysis of the hind limbs, or convulsions) [20].

According to research conducted by Yin *et al*. [21], cats suspected of having FIPV exhibit clinical symptoms, such as pleural effusion; some also display ascites, weight loss, lethargy, and lack of appetite. Jaundice and fever are additional clinical symptoms associated with the virus [22]. The study results of Moyadee *et al*. [18] indicated that FIP-affected cats predominantly presented with symptoms, including abdominal distension, depression, dehydration, anorexia, dyspnea, jaundice, diarrhea, and vomiting. These cats exhibited decreased levels of blood urea nitrogen, creatinine, and albumin but increased levels of globulin relative to the reference interval. FIP can manifest as two primary clinical forms – wet and dry – that may sometimes overlap. The wet form is characterized chiefly by the accumulation of protein-rich fibrin fluid in the body cavity, which leads to symptoms indicative of severe and acute hypovolemia and/or organ compression within the affected cavity, such as dyspnea and reduced peristalsis. Conversely, the clinical manifestations of dry form depend primarily on the site of granulomatous lesion formation, most often the kidneys, resulting in clinical and laboratory signs of renal dysfunction. However, extra-renal symptoms may also manifest if granulomatous lesions are widespread in other organs, such as the liver, lungs, intestines, and notably, the eyes and central nervous system (CNS) [23].

According to a previous study by Crawford *et al*. [24], the clinical symptoms observed in cats with FIP include ataxia, lack of appetite, head tilt, head tremors, and seizures. Conversely, the physical examination revealed a thin body, abdominal distension, dehydration, and pallor. Neurological examination showed head tremors, head tilt, ambulatory tetraparesis, vestibular ataxia, and ambulatory paraparesis. Three distinct neurological syndromes were identified: T3-L3 myelopathy without obvious brain involvement, central vestibular disease, and multifocal CNS disease accompanied by tetraparesis.

**Figure-1:**
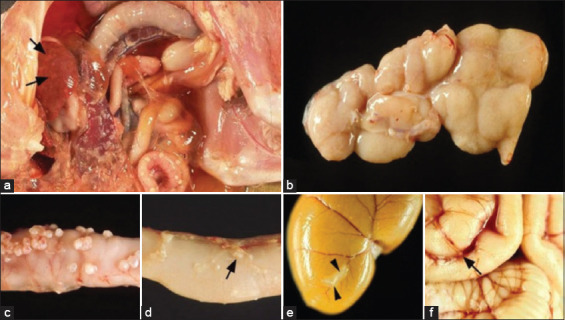
(a–f) Gross lesions in feline infectious peritonitis (FIP). Wet FIP is represented by serofibrinous and granulomatous serositis and granulomatous lesions in the liver (arrows). (b–f): Cats with dry FIP. Enlargement of mesenteric lymph nodes due to granulomatous inflammation. Jejunum with multiple granulomas in the serosa. Jejunum with small subserous granulomatous lesions in the veins (phlebitis and/or periphlebitis; arrows). Kidney with granulomatous and periphlebitis of the capsular vein (arrow). (f): Brain with multifocal granulomatous phlebitis and periphlebitis of the cortical leptomeningeal vein (arrow) [25].

**Figure-2:**
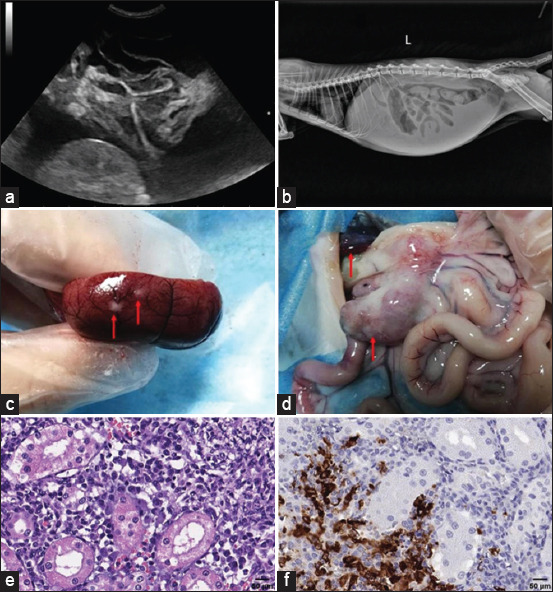
(a) Ultrasound image of ascites. (b) Radiograph of a large amount of effusion in the abdominal cavity. (c) Presence of necrotic foci in the kidneys. (d) Ascites and enlarged lymph nodes. (e) Interstitial nephritis is characterized by inflammatory cell infiltration, including macrophages, lymphocytes, neutrophils, and plasma. (f) Presence of macrophages in the kidney using immunohistochemical staining [21].

**Figure-3:**
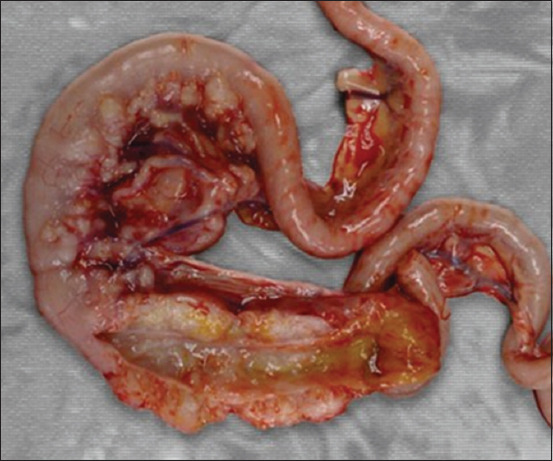
Feline small intestine with extensive thickening of the intestinal wall characterized by dense, white, irregularly proliferating tissue that extends through the intestinal wall [26].

**Figure-4:**
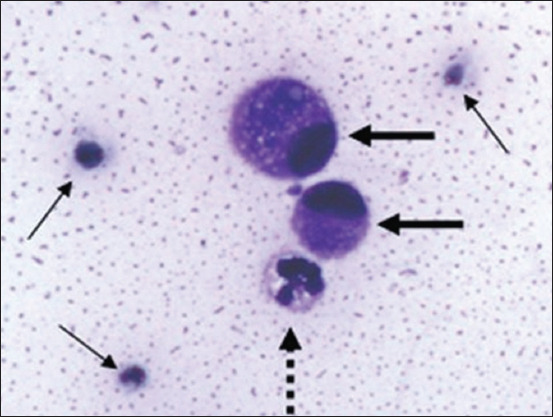
Abdominal effusion from a feline infectious peritonitis cat. Non-degenerated neutrophils with vacuolated hyperbasophilic cytoplasm (dashed arrow), two mesothelial cells (thick arrow), and scattered erythrocytes (thin arrow) embedded in a granular proteinaceous eosinophilic background [26].

**Figure-5:**
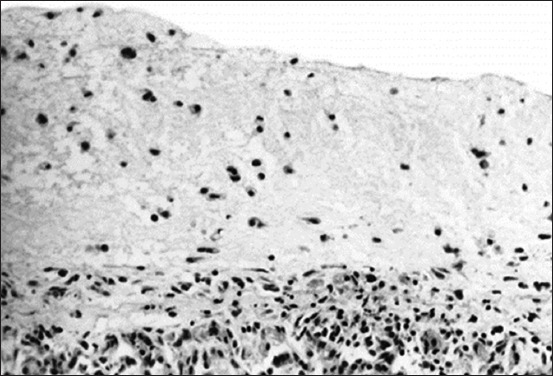
Cat lung organ with severe, acute, diffuse fibrinous pleuritis accompanied by scattered inflammatory cells [22].

**Figure-6:**
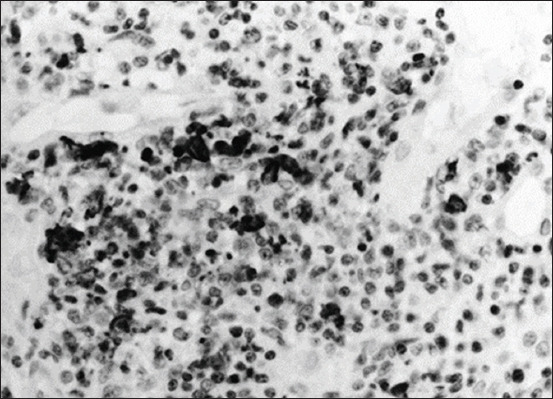
Feline liver pyogranulomatous hepatitis with intracellular positivity [22].

**Figure-7:**
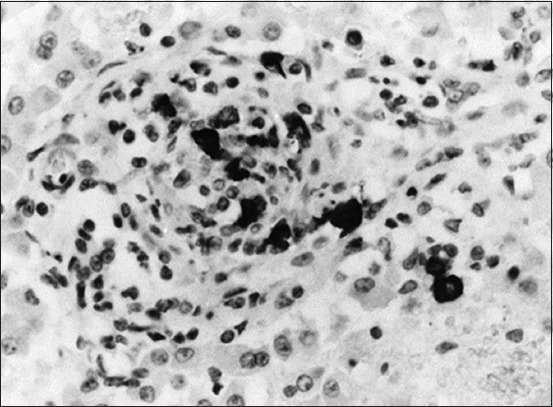
Kidney organs of feline infectious peritonitis cats experiencing granulomatous nephritis with positive presence of intracellular and extracellular granules [22].

Figure-1 presents gross lesions of FIP [25]. Figure-2 presents an ultrasound image of ascites, a radiograph of a large amount of effusion in the abdominal cavity, necrotic foci in the kidneys, ascites and enlarged lymph nodes, and interstitial nephritis [21]. Figure-3 presents feline small intestine with extensive thickening of the intestinal wall characterized by dense, white, irregularly proliferating tissue that extends through the intestinal wall [26]. Figure-4 presents abdominal effusion from a feline infectious peritonitis cat. [26]. Figure-5 presents cat lung organ with severe, acute, diffuse fibrinous pleuritis [23].Figure-6 presents feline liver pyogranulomatous hepatitis with intracellular positivity [23]. Figure-7 presents kidney organs of FIP cats experiencing granulomatous nephritis with positive presence of intracellular and extracellular granules [23].

**Figure-8:**
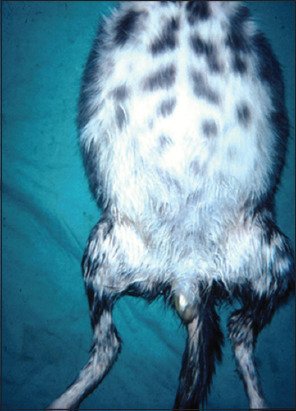
The kitten’s stomach is very distended and accompanied by effusive feline infectious peritonitis. There is also an enlarged scrotum due to inflammation of the tunica [16].

Notably, some clinical symptoms are uncommon, such as those affecting the male genitalia – specifically, scrotal enlargement due to peritonitis spreading to the tunica surrounding the testicles and resulting in edema (Figure-8) [16]. In most cases, all clinical signs were identified simultaneously; however, there were instances in which neurological involvement was not clear until the later stages of the disease. Although most cats exhibited symptoms indicating a widespread CNS disease, three showed neurological abnormalities, suggesting localized involvement. The most commonly observed neurological symptoms were convulsions, nystagmus, and posterior paresis. All cats either died in the hospital or were euthanized at the owner’s request. Most affected cats exhibited not only neurological and visual impairments but also signs of systemic involvement. Medical histories and physical examinations often revealed one or more of the following findings: Fever, palpably enlarged lobulated kidneys, pale mucous membranes, despondency, and anorexia [27].

**Figure-9:**
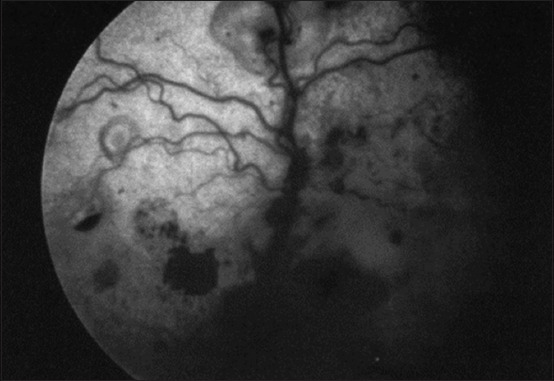
Feline infectious peritonitis-related retinal hemorrhage and detachment [29].

**Figure-10:**
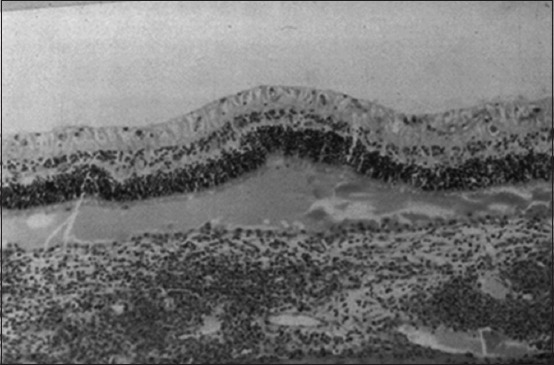
Histopathologic slice of the eye. Mononuclear infiltration in the choroid and exudative retinal detachment are visible as fluid in the subretinal gap separating the retina from the choroid [29].

**Figure-11:**
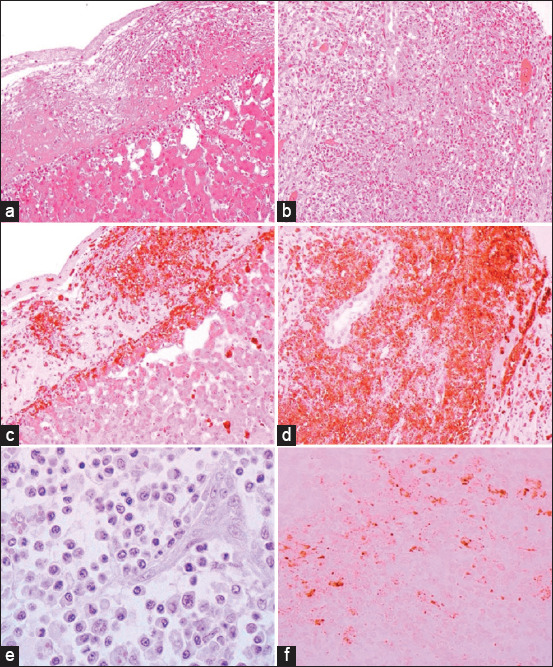
Histopathological and immunohistochemical characterization of feline infectious peritonitis lesions. (a) Fibrous exudation with diffuse serosal inflammation. (b) Renal granuloma development (type B lesion). (c) Numerous red cells or monocytes/macrophages in the same inflammatory region, as seen in (a). (d) Numerous monocytes/macrophages (red cells) within the same granuloma, as shown in (b). (e) Plasma cells and a multinucleated giant cell (upper right corner) within a type A lesion in the liver (cat no. 6). (f) Inflammatory cells (presumably macrophages) containing feline coronavirus (FCoV) antigen (red cells) within an intestinal granuloma. FCoV-positive granules are also present extracellularly. Stainings: (a, b, and e): hematoxylin-eosin, (c and d): lectin immunohistochemistry, and (f): FCoV immunohistochemistry. Magnifications: (a–d): 100×, (e): 400×, and (f): 200× [30].

Ocular manifestations of FIP are significantly more common, and in certain cats, they are the initial complaint. Specifically, the blood-aqueous barrier breaks down, causing fibrin to leak into the anterior chamber from vein blood vessels. Exudation of either white blood cells (hypopyon) or red blood cells (hyphema) into the anterior chamber is indicative of uveitis and a significant breakdown of the blood-aqueous barrier. Miosis, or constriction of the pupil, is caused by stimulation of the iris sphincter muscle after prostaglandin release during uveitis. The complaint may be a subtle or noticeable alteration in the color of the iris, particularly in patients with chronic anterior uveitis. Notably, ocular hypotony is caused by a decrease in aqueous humor output, resulting in lower intraocular pressure than normal [28]. Figure-9 shows FIP-related retinal hemorrhage and detachment [29]. Mononuclear infiltration in the choroid and exudative retinal detachment is visible as fluid in the subretinal gap separating the retina from the choroid (Figure-10) [29], Histopathological and immunohistochemical characterization of feline infectious peritonitis lesions, Fibrous exudation with diffuse serosal inflammation, Renal granuloma development, Numerous red cells or monocytes/macrophages in the same inflammatory region as seen in, Numerous monocytes/macrophages (red cells) within the same granuloma as shown in, Plasma cells and a multinucleated giant cell (upper right corner) within a type A lesion in the liver, Inflammatory cells (presumably macrophages) containing feline coronavirus (FCoV) antigen (red cells) within an intestinal granuloma. FCoV-positive granules are also present extracellularly (Figure-11) [30].

The classic features of FIP include the onset of effusion in the abdominal or thoracic cavity. In addition, it presents clinical symptoms of endothelial dysfunction and vasculitis, with lesions characterized by edema and perivascular infiltration, vessel wall degeneration, and endothelial proliferation [28].

**Figure-12:**
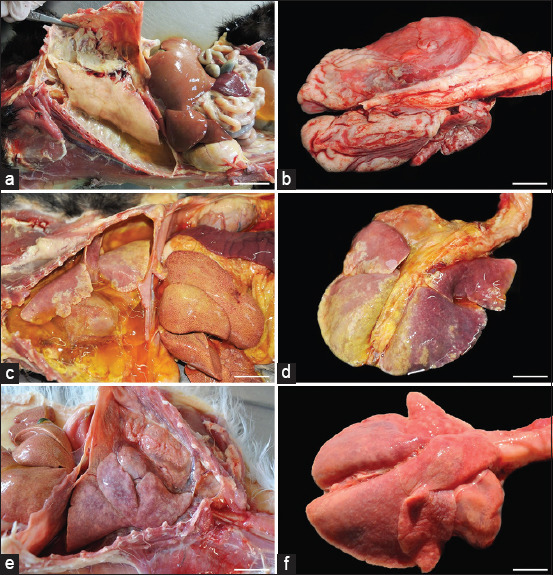
(a-f) Feline infectious peritonitis in the lungs of cats [28].

In the lungs, three primary gross patterns of damage were observed. Of the 66 cats studied, 18 exhibited a yellow to reddish liquid, fibrillar, gelatinous, or pasty substance in their thoracic cavities that adhered to both the parietal and visceral pleura. Importantly, there were no inflammatory parenchymal changes, and the pulmonary parenchyma was irregularly pleural and diffusely atelectasis (Figures 12a and b) [28]. Similarly, the visceral and parietal pleura of the 13 cats exhibited a noticeable deposit of similar yellow liquid, fibrillar, gelatinous, or pasty material. In these cases, the lung parenchyma often remained intact, featuring many white nodules sporadically spaced a few millimeters in diameter throughout the lung lobes (Figures-12c and d) [28]. Furthermore, in 23 additional animals, there was little to no accumulation of yellowish fibrillar debris in the thoracic cavity. Some showed impressions of the ribs, and the lung lobes were not deflated. Notably, the lung parenchyma frequently exhibited a noticeable pallor and several sporadically scattered white nodules, each a few millimeters in diameter (Figures-12e and f) [28].

## Pathogenesis

The pathogenesis of FIP involves immune complex formation and Ab-dependent enhancement of viral infections. This study observed vasculitis and immune complex deposition. Clinically, effusive (wet) and non-effusive (dry) manifestations of FIP are evident. The effusive type is characterized by fluid accumulation in bodily cavities, fibrinous pleuritis, and peritonitis. In the non-effusive form, pyogranuloma may develop in the eyes, lungs, CNS, and abdominal viscera. Approximately 75% of clinical cases were effusive, whereas the remaining 25% were non-effusive. Some cats exhibited symptoms consistent with both types of FIP. An exuberant form arises when there is a strong host humoral immune response and a weak cell-mediated immunological response. Conversely, a non-effusive form develops in response to an inflammatory response [31]. Viral factors are critical in the pathophysiology of FIP. It has been established that the S glycoprotein of FCoV controls host cell entry, and mutations in the S gene affect cell tropism. Comparisons between FCoV excreted in the feces of clinically “healthy” cats and FCoV from FIP tissues reveal more frequent mutations at various positions within the S gene. This observation led to the hypothesis that some of these mutations may serve as useful markers for distinguishing between cats with and without FIP. Nevertheless, a recent comprehensive study indicated that one of these mutations, related to a fusion peptide, is more indicative of systemic FCoV infection than FIP itself. Systemic FCoV infection was observed in FCoV-viremic cats, both with and without FIP, at equal frequencies [32].

Target macrophages and monocytes are exposed to FIPV through initial surface binding, followed by internalization through clathrin- and caveolae-independent and dynamin-dependent endocytosis [16]. The most important step in the viral life cycle is virus entry into the cell. In FIPV, viral entry into cells occurs through two mechanisms. The first pathway involves cytosolic entry through early endosomes (FCoV-II) and the second through late endosomes (FCoV-I). Hartman *et al*. [33] revealed that the cellular receptor of FCoV-II is aminopeptidase N (APN), which binds to S proteins and mediates viral internalization into cells. However, no relevant studies have investigated FCoV-I cell receptors. FCoV binds to these receptors and requires cytoplasmic access for replication. The S protein is cleaved by cathepsin B and fuses with the endosomal membrane following endocytosis of the complex between the viral receptor and viral S protein. The fusion peptide of the S protein is located in the S2 domain. FCoV-I possesses two specific activation sites: S1/S2, which is cleaved by furin-like protease and S2’, which is cleaved by cathepsin B. FCoV-II contains only the S2’ site, which is cleaved by cathepsin B. Cathepsin B is likely the most crucial protease facilitating FCoV entry, with cathepsin L playing a probable secondary role. FCoV replication occurs rapidly, and the cycle is completed in <24 h. Mutations in the FCoV S gene contribute to the shift in cell tropism and pathogenicity. Following oral-fecal transmission, FCoV initially infects the intestinal tract and may disseminate to other areas, causing monocyte-associated viremia [33].

These antigen-Ab complexes are believed to be identified by macrophages but are not delivered to killer cells as they should be, preventing them from being eliminated. The outcomes of immunological complex formation in cats depend on the antigen content, Ab concentration, and size of the complex. Moreover, immune complex deposition most likely occurs at blood vessel bifurcations, which are locations of high blood pressure and turbulence. FIP lesions are frequently found in the kidney, uvea, and peritoneum and are characterized by turbulence and elevated blood pressure [34]. There is no information on the precise viral genetic factors associated with FIPV pathogenesis. According to the *in vivo* mutation transition theory, virulence develops through *de novo* virus mutations occurring *in vivo*. A previous study by Brown *et al*. [35] indicated that sequence variations in the S protein, non-structural protein (NSP) 7b, and NSP3c are disease determinants; however, the specific type of mutation causing disease has not yet been identified. FIP is a systemic illness that manifests as either “wet” or effusive FIP, characterized by cavitary effusions, or as “dry” or non-effusive FIP, characterized by granulomatous lesions and/or vasculitis. These two pathotypes have long been considered independent viral species.

Nevertheless, molecular research has demonstrated that they are two virulence-varying forms of the same virus. Consequently, variations in intestinal strains have been proposed as responsible for the differing pathogenicity. No mutation has been identified as a definitive cause of FIP, although several potential genes have been linked to this virulence shift. Irrespective of virulence, a recent study by Tasker *et al*. [7] has shown that FCoV S protein mutations indicate systemic dissemination of the virus.

After a few days, experimental cats infected with either the prototype serotype II FIPV 79-1146 strain or a recombinant variant of this virus developed fever and rapidly lost weight. Viral RNA was detected in the feces and blood early after infection, and serum Ab titers increased rapidly, remaining high throughout the illness. In other cases, diseased cats appeared to recover after the first week [36]. A previous study by Chang *et al*. [37] on the pathophysiology of FIP has focused on the S gene. The binding of receptors and entry of the virus depend on the coronaviral S protein. The biotype flip may result from mutations in S gene alone or in conjunction with alterations in other genes because the FECV-FIPV transition involves a shift in target cell tropism. Recent research has explored the role of S gene mutations in the pathophysiology of FIP to address this issue. Most FIPVs can be distinguished from FECVs by two-point mutations in the S gene, according to an analysis of 11 full-length genome sequences for FECV and FIPV [37].

## Hematology

*Coronaviridae* possess a single-stranded RNA genome. There are two serotypes in the FCoV division; Serotype I is the serotype found in cats. Strong pleocytosis (>100 cells/mL), high protein concentrations (>200 mg/dL), and FCoV Ab titers of >1:25 were observed in an analysis of FIP-infected cats [38]. Serotype II is the evolutionary result of a recombination event between FCoV and canine enteric coronavirus, resulting in a chimeric FCoV encoding the canine coronavirus spike gene [38].Macrophages, the primary FIPV target cells, may penetrate the mucosal barrier and disseminate the virus throughout the cat. Moreover, a correlation was observed between the *in vivo* virulence of FIPV and its ability to affect macrophages *in vitro*. It has been proposed that humoral immunity may not be as effective in defending cats against FIPV as robust cell-mediated immune responses [39].

FIP often leads to hematological irregularities, including lymphopenia, neutrophilia, anemia, and thrombocytopenia. Moreover, serum biochemical anomalies commonly associated with FIP include hyperproteinemia, hyperbilirubinemia, hyperglobulinemia, and hypoalbuminemia. Hyperglobulinemia is also observed in conditions such as lymphoma, multiple myeloma, and persistent infections [40]. When cats with FIP exhibit hyperglobulinemia (mostly gamma globulins), hypoalbuminemia (primarily hypoalbuminemia), or both, hyperproteinemia frequently manifests as a common laboratory anomaly. Moyadee *et al*. [41] identified hyperglobulinemia and hypoalbuminemia in cats with FIP, but no increase in total protein levels was observed. Only 17.5% of cats with FIP exhibit hyperproteinemia, and effusive FIP is less likely than non-effusive FIP to increase total blood protein [41]. Hyperglobulinemia may occur independently or concurrently with hypoalbuminemia and hyperproteinemia. Hypoalbuminemia and hyperbilirubinemia are frequently associated with effusion, whereas azotemia is common in cats without effusion [42]. Clinically, affected patients may exhibit various symptoms depending on the organ system involved. These include elevated serum levels of liver enzymes and bilirubin, increased serum urea nitrogen and creatinine, elevated fibrinogen levels, reduced packed cell volumes, neutrophilia, lymphopenia, and proteinuria. When FIP affects the CNS, cerebrospinal fluid (CSF) analysis often shows increased cellularity and protein content [3]. Ab testing does not differentiate between FECV and FIPV, and Ab titers are unreliable indicators of FIP. Although most cats have Abs against FCoV, they do not necessarily develop FIP. Importantly, Abs against the FCoV 7b protein can be detected, assuming that FIPV includes the 7b gene [42]. Typically, effusion tests offer more accurate predictive values than blood tests [23]. Clinical pathological histories, such as full blood and serum biochemical profiles and investigations of effusion or CSF are essential for FIP diagnosis [43].

Blood samples from cats were drawn into vacuum blood collection tubes without anticoagulant, stored at 4°C overnight, and centrifuged for 10 min at 1000× *g* [44]. The serum was then kept cold until further use. The Antech Veterinary Diagnostic Laboratory employs an automated cell counter to evaluate fresh whole blood and complete blood counts [23]. The failure to directly detect FIPV antigens in blood using Abs may be due to low levels of viremia [45]. Serum chemistry tests play a crucial role in the detection of FCoV during various infection phases. Cats showing the clinical signs of infectious peritonitis underwent polymerase chain reaction testing. Both serum chemistry tests and the EvaGreen real-time reverse transcription polymerase chain reaction (RT-PCR) assay were used to detect FIPV, which leads to hyperglobulinemia. A real-time PCR test using EvaGreen targeting the highly conserved N gene was developed to screen for FCoV in cats. For a definitive diagnosis, viral antigens in cat monocytes must be stained with immunohistochemical dye [44]. Pedersen [8] has shown that FIP cats exhibit a higher ratio of peripheral blood surface immunoglobulin-positive cells to CD21+ cells than specific-pathogen-free cats. Furthermore, an increase in the number of cells expressing the plasma cell master gene encoding B lymphocyte-induced maturation protein is noted [8].

## Clinicopathological and Imaging Features

The two biotypes of FCoV are FECV and FIPV. Both biotypes are pathogenic; however, they differ in their infection mechanisms in cats and their potential to cause FIP [40]. FIP is a serological disease that is challenging to differentiate genetically from FECV; thus, further diagnostic tools are required [23]. Despite the similarities in the epidemiological and virological characteristics of these two infections, the pathophysiology of the illness distinctly differs from that of severe acute respiratory syndrome coronavirus 2 (SARS-CoV-2). This difference starts at the cellular entry level, which is prompted by binding SARS-CoV-2 to the human angiotensin-converting enzyme 2 (ACE2) receptor [22]. Additional diagnostic approaches include the use of magnetic resonance imaging (MRI) in cats exhibiting neurological symptoms of FIP [23]. MRI can identify FIP through signs of ventricular dilatation in cats, a condition known as ventriculomegaly [46]. The extent of contrast enhancement variations correlates with the degree of ventricular dilatation. Epididymitis, multifocal hepatitis, and secondary obstructive hydrocephalus have also been observed in MRI findings [47].

**Figure-13:**
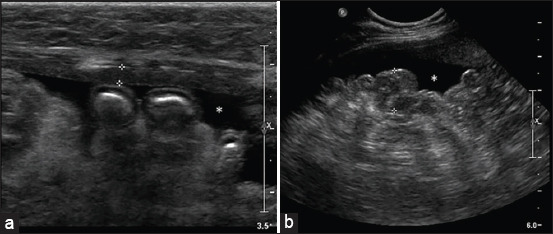
Feline infectious peritonitis in a 12-year-old female cat: a longitudinal ultrasound image of the abdomen (case 9). (a) Between the calipers, a slightly thickened section of the parietal peritoneum is visible (2.6 mm). (b) The hyperechoic mesentery presents an uneven and nodular appearance (between calipers: 1.2 cm). Furthermore, an anechoic peritoneal effusion (asterisk) is observed [48].

Abdominal ultrasound is used to detect potential abnormalities, including abdominal effusion (noted in 75% of cases) and irregularities in the kidneys (69%), lymph nodes (56%), and/or liver (37%) [48]. Feline infectious peritonitis in a 12-year-old female cat showed an anechoic peritoneal effusion (Figure-13) [48].

Leukopenia may accompany diarrhea. The small intestinal epithelium is a target of FECV [8]. Staining of the spleen, liver, brain, lymph nodes, lungs, gut, and kidney slides with hematoxylin-eosin reveals granulomatous and pyogranulomatous lesions [49]. In the context of ascites etiology in cats, abdominal distension is more common than neoplasia, cardiovascular disease, or hepatic or renal disease, and it is the most frequent physical finding in cases of wet FIP. Upon abdominal exploration, up to 1 L of yellow-tinged, slightly to moderately cloudy mucinous fluid is typically found [16].

Brain lesions are a consequence of FIP infection, including either meningoencephalitis or meningomyelitis. Stomach lesions are prevalent in the intestine [43]. Spinal cord abnormalities were detected by MRI of the patient’s brain. Observable abnormalities include ventriculomegaly, which is characterized by enlarged ventricles in cats. Periventricular hyperintensity indicates interstitial edema. The significant aspects of this imaging procedure include cerebellar herniation, ventricular dilatation, and meningeal and ependymal contrast enhancement, even when obstructive hydrocephalus is highly improbable [24]. Hydrocephalus, which arises from ependyma and choroid pathologies, has been recorded and may lead to convulsive disorders, dementia, or personality changes, such as rage, aggression, hiding, or withdrawal. Vestibular cerebellar symptoms, such as circling, head tilting, and nystagmus, also arise from FIP [16]. The clinical manifestations of CNS involvement in cats with dry FIP differ according to severity, specific location within the nervous system, and organ involvement [16]. FIPV is mutagenic and can disseminate through the bloodstream. Both effusive and granulomatous multisystemic inflammatory responses are triggered by the replication of this mutation in macrophages. In patients with granulomatous FIP, the body cavity lacks an inflammatory exudate. Granulomatous FIP is a distinct clinical manifestation of the exuberant form. Small granulomas form around the arteries and venules of the CNS in this type, affecting the CNS. The virus induces a cellular inflammatory response around CNS arteries and venules, leading to the formation of tiny granulomas. Periventricular vasculitis results in periventricular reactive astrocytosis and exudation of cells and proteins [50].

There is only one published report by Park *et al*. [51] on a cat diagnosed with diffuse lung consolidation, pyogranulomatous pneumonia, and non-effusive FIP. In this case, numerous masses were noted in the pleura, lung, and kidney, along with recurrent granulomatous changes. Cats with the effusive form of FIP often develop small bowel disease. Typically, the radiographic hallmark of pneumonia includes alveolar infiltrates on air bronchogram; however, pneumonia resulting from bite wounds or foreign bodies may also produce mass-like lesions adjacent to the lung or pleural wall [51]. Common symptoms in kittens include pneumonia, pleurisy, and hepatitis. In cats with FIPV, immune complex formation or migration of macrophages/monocytes into the synovium, leading to generalized synovitis [16]. Definitive confirmation of FIP necessitates the detection of the virus within lesions or effusions. Certain clinical or clinicopathological findings strongly suggest the presence of FIP [23].

To support the diagnosis of non-effusive FIP, RT-PCR can be used alongside biochemical, serological, and hematological assessments [52]. FIPV cannot be conclusively identified using serological methods alone; hence, hematological and serum biochemical tests are also performed. The RT-PCR method can be used to diagnose FIPV. Suitable specimens include body cavity fluid (a cyst and pleural effusion), CSF, blood, and tissue. Using CSF can lead to highly specific RNA detection [53]. Tissues suitable for RT-PCR analysis include liver, kidney, and spleen samples [54].

## Pathological Features

FIP is a systemic illness characterized by granulomatous lesions (“dry” or non-effusive FIP) and/or vascular disease. This may lead to cavitary effusions (often called “wet” or effusive FIP) [22]. Effusive FIP, the more traditional and prevalent form, typically progresses rapidly, accumulating fluid in the peritoneal and thoracic cavities. Conversely, the “dry” or granulomatous variant has a more insidious onset with extensive granuloma formation across multiple organs rather than cavitary effusion [55]. The symptoms of FECV infection can emerge anywhere from 10 to 15 days to several months after infection. The disease progresses from a subclinical state to a clinical state without improvement [56]. The lesions include hepatitis/capsulitis, pancreatitis, ascites or abdominal effusion, serositis/leiomyositis, lymphadenitis, jaundice, perivasculitis, and uveitis. In addition, necrotizing interstitial nephritis has been noted in the kidneys [57]. Natural occurrences include nodules on the renal medulla surface and coarse lesions with grayish-white patches. Detachment of the parenchyma from the renal capsule poses a challenge. Variability in granule size has been noted in the brain. The lesions observed were parts of the third and fourth ventricles which were lateral ependyma and leptomeningea. The inner layers of the cornea, iris, ciliary bodies, choroid membrane, and occasionally the retina exhibit signs of cerebral changes. Experimental autopsies revealed small focal clusters of neutrophils and large mononuclear cells. Within the granulomas, large mononuclear cells, along with neutrophils, lymphocytes, plasma cells, and fibrin, are present and show central necrosis [58].

Pneumonia is not a common characteristic of FIP. The presence of pneumonic lesions indicates the potential for simultaneous infection by microorganisms. For example, groups of coccoid and Gram-negative bacteria in aggregate form may be distributed in the respiratory tract and cause inflammation in affected cats. The collection of bacteria and foreign bodies may be observed in the bronchi, often accompanied by aspiration pneumonia [38]. Furthermore, necrosis in the stellate and arcuate veins was observed in the kidneys, along with intimal neutrophil aggregation and granulomatous lesions, suggesting perivascular lesion development. An early-phase granulomatous lesion was identified. Granuloma formation in intralobular venous adventitia is evident in lung lesions. Small blood vessels have been observed in the brain and leptomeninges [58]. The following pathological features were obtained from the typical gross postmortem observations of FIP cases. Granulomatous lesions, organ serosa, and fibrinous plaques can be observed within the thoracic or abdominal cavity. The mesenteric lymph nodes, liver, spleen, kidneys, intestinal surfaces, diaphragm, and wall of the abdominal peritoneum were examined. In severe cases, the peritoneal and/or pleural cavities may contain yellowish, viscous fluid, and fluid accumulation may also be noticeable in the pericardium [59].

## Experimental Infection

In an experimental setting, cell fusion was conducted 6 times using mouse spleen cells immunized with the FIPV strain 79-1146. Seven monoclonal Abs (mAbs) capable of neutralizing FIPV strain 79-1146 were isolated and designated 5-6-2, 5-7-2, 6-1-1, 6-4-2, 7-1-1, 7-3-1, and 7-4-1. The polypeptide specificity of these Abs was determined by western blotting. A competitive protection test was conducted to investigate variations in epitope specificity among the seven neutralizing Abs. Furthermore, the reactivity of the mAbs that recognize the three neutralizing epitopes in FIPV strain 79-1146 with feline, swine, and canine coronaviruses within the same class was assessed using a neutralization test (NT). These mAbs did not neutralize the six FIPV type I strains but did neutralize the FECV 79-1683 strain, a swine and canine coronavirus. Notably, the mAbs targeting epitope III (6-1-1 and 6-4-2) demonstrated excellent neutralizing efficacy against this virus, including FIPV strain 79-1146. In addition, the NT was used to evaluate the reactivity of three neutralizing mAbs (55-2, 38-1, and 66-A) generated using the transmissible gastroenteritis virus (TGEV) strain TO-163 as an immunogen against cat, dog, and swine coronaviruses. All three mAbs recognize distinct epitopes of the TGEV S protein [60]. Viruses were detected in the oropharyngeal secretions and feces of all FIPV-inoculated cats. In all cases, viral shedding was observed in the oropharynx on the 2^nd^ day after infection and persisted until the 9^th^ or 10^th^ day. Most animals had a brief second oropharyngeal discharge on day 14 in conjunction with the emergence of clinical symptoms. Cats infected with FIPV exhibited a rapid increase in Ab titers in the indirect immunofluorescent straining technique (IFT) and serum neutralization (SN) tests, regardless of the mode of inoculation. The SN test revealed an Ab titer > 2000 on day 9. However, this threshold was not reached in the IFT when most cats succumbed (day 18), although the titers continued to increase. Cats that survived had titers comparable to those of the other cats in the SN and IFT tests. Before inoculation with a virus or cell lysate, the responses of cats to ConA vary from week to week [61].

Pedersen *et al*. [62] used several FIPV strains, such as FIPV-UCD1, -UCD2, -UCD-3, and -UCD4. Cats infected with FIP-UCD2, -UCD3, and -UCD4 were administered 1 mL tissue culture fluid from *Felis catus* whole fetus (Fcwf)-4 cells infected with the fifth-passage virus. The inoculation route included either oronasal (1/2 mL orally and 1/2 mL intranasal) or intraperitoneal administration. Additional FIPV strains were also introduced into infected cats, as previously documented. Cell immunity to FIPV can be assessed in two ways: (1) by observing distinct lymphocyte growth in response to the FIPV antigen and (2) by evaluating a delayed-type hypersensitivity reaction to FIPV. The FIPV-79-1146 and Nor15 strains exhibited similar levels of contagiousness and were transmitted through both oral and parenteral routes of exposure. However, isolates, such as FIPV-UCD3 and -UCD4, demonstrated similar transmission effects when administered orally or intraperitoneally but showed significantly higher virulence when delivered orally. In contrast, FIPV-UCD1, which is known for its relative malignancy, displayed lower infectivity when administered orally than when administered parenterally. Despite being extremely contagious through oral and intraperitoneal routes, FIPV-UCD2 has lost virulence. Hence, virulence and infectivity are distinct factors that operate independently [62].

## Treatment and Prevention

FIP is a global disease that affects domestic and wild felids. Although much research has been conducted on FIP, it remains one of the most prevalent and fatal infectious diseases in cats [21]. As clinical indications worsen, compassionate euthanasia in shelter settings is a suitable method to end suffering [63]. The immunosuppressive macromolecule cyclosporine A (CsA) interacts with chaperone proteins known as cyclophilin, facilitating the cis/trans conformational shift of proline residues. The interaction of CsA with abundant cellular cyclophilin likely contributes to its antiviral effect. CsA exhibited dose-dependent cytotoxicity at high concentrations (cytotoxic concentration of 50% [CC_50_] of 14.1 μM) and inhibited FIPV in fwcf-4 cells at concentrations from 0.16 to 10 μM during post-infection therapy. CsA therapy was administered to a client-owned cat suffering from severe FIP, with dosages ranging from 25 mg/kg to 75 mg/kg. The cat died of respiratory failure 264 days after the initiation of therapy. The symptoms and effusion initially subsided, but relapse occurred on day 251 of treatment [64]. Historically, FIP was regarded as a progressive, fatal disease; however, with the development of new antiviral agents, such as nucleoside analogs and protease inhibitors, there are now views that FIP might be a treatable disease. It is estimated that some cats may locally transmit the disease for months or even years [65]. Cats diagnosed with advanced FIP often exhibit compromised immunity and high viral counts, and the use of glucocorticoid therapy may intensify this condition. Although these medications are crucial for managing the immune response associated with FIP, they may render individuals susceptible to bacterial infections through overall immunosuppression and myelosuppression. Therefore, broad-spectrum antibiotics are required for preventive purposes. Therefore, amoxicillin and cefadroxil are considered viable alternatives. In cases of confirmed infection, antibiotics should be selected based on culture results and sensitivity tests [66]. Supportive therapy entails the use of appetite enhancers (such as mirtazapine, up to 2 mg/cat/day), vitamin B12 supplements (administered weekly through subcutaneous injection at 0.002 mg/kg or orally at 0.25 mg/cat daily), antioxidants, and hydration therapy. The efficacy of effusion drying remains an ongoing topic [65].

The use of therapeutic abdominocentesis remains controversial due to the potential harm caused by the removal of substantial and rapidly fluctuating fluid volumes. In some instances, treatment was performed concurrently with intracavitary steroid administration, specifically dexamethasone at a dose of 1 mg/kg once daily until resolution or for a maximum of 7 days, in conjunction with other treatments. Temporary resolution of effusion was observed in 6 of 36 cats; however, all cats eventually succumbed to FIP [67]. Ribavirin, also known as 1-β-D-ribofuranosyl-1H-1,2,4-triazole-3-carboxamide, is a broad-spectrum triazole nucleoside that is notable for its antiviral activity against both FCoV and other RNA and DNA viruses. Unlike most conventional antiviral drugs, which primarily inhibit polymerase, this nucleoside analog permits DNA and RNA synthesis but likely interferes with viral messenger RNA (mRNA) capping, inhibiting viral protein production. Therapeutic doses are challenging to produce *in vivo* due to toxicity, and cats are particularly susceptible to adverse effects. Although ribavirin is active against FCoV *in vitro*, it is ineffective against FIP in cats. Hartman *et al*. [34] administered ribavirin (16.5 mg/kg orally, intramuscularly, or intravenously every 24 h for 10–14 days) to select kittens free of pathogens 18 h after a viral trial that induced FIP. The FIP killed all of the kittens, including those treated with ribavirin and those left untreated. The clinical signs of illness were more severe in kittens treated with ribavirin, and their average survival time was shorter. Hemolysis is the most common adverse reaction in cats, as documented in a study by *Hartman et al*. [34] (including those using 11 mg/kg). This phenomenon is attributed to the sequestration of medications by red blood cells. In addition, harmful dose-related effects occur in the bone marrow, particularly affecting megakaryocytes (leading to thrombocytopenia and bleeding) and erythroid precursors [34]. Prednisolone is commonly used to alleviate symptoms associated with chronic inflammation despite the lack of clinical trials supporting its use. An initial oral dose of 0.5 mg/kg twice daily is recommended orally. A previous study by Meli *et al*. [68] indicated that cats with non-effusive FIP had a considerably shorter survival duration than those treated with corticosteroids or immunostimulants [65]. Cats with FIP can be treated using various techniques. Medications that specifically inhibit viral replication have demonstrated efficacy against various viral conditions, such as human immunodeficiency virus type 1 (HIV-1), hepatitis B, and hepatitis C viruses. Another approach involves impeding key elements of the inflammatory response using drugs, such as interferons. When interferon-α is combined with antiviral medications, such as tenofovir, entecavir, and ribavirin, it can effectively combat hepatitis B and/or C virus infections. However, reliance solely on this approach seldom yields success. The third strategy involves non-specific strengthening of the immune system to overcome infection. Some treatments involve the combination of one or more therapeutic methods. Irrespective of the chosen approach, rigorous clinical trials assessing safety and efficacy should be conducted for any article in a scientific journal that asserts the efficacy of therapies for FIP [8].

Antiviral medications are categorized into two main types: Those that target the cellular machinery of viruses that rely on replication assistance and those that focus on specific processes during viral infection and replication. Drugs that target cellular systems tend to be less effective because they can harm both the host and the virus. While prednisolone and alkylating medications, such as cyclophosphamide, have been used to alleviate clinical symptoms in cats with FIP, limited evidence supports their efficacy in improving illness outcomes. Rather than resorting to less focused treatment approaches, efforts have been made to suppress specific cytokines believed to play crucial roles in FIP development. Tumor necrosis factor inhibitors have been used to alleviate the pain symptoms associated with FIP [69]. The most effective antiviral drugs target specific segments of the viral genome to control critical processes during infection or replication. FCoV shares numerous genes with functions analogous to HIV-1, including RNA virus-dependent polymerases and proteases. Retroviral proteases are vital targets of HIV-1, and the combination of reverse transcriptase, protease, and integrase inhibitors has significantly transitioned HIV-1 into chronic subclinical infections in many patients. Building on the successes of other viral protease inhibitors, similar medications targeting the main protease (3CL) encoded by coronaviruses and noroviruses are currently under development [8]. Cytokines have been used to modulate immune responses with inconsistent results. Recombinant human and feline interferon has no significant effect in cats with FIP. The immunostimulant polyprenyl, which enhances T lymphocyte response to induce cell-mediated immunity in cats, has shown mixed success. Its mechanism of action, however, remains unclear. Notably, successful dry-type FIP has improved the survival of infected cats. Conversely, this success has not been replicated in cats with wet-type FIP. In a field study using this chemical, 8 of 60 cats with FIP survived for more than 200 days, with 4 exceeding 300 days; all affected cats had dry-type FIP [70].

Prospectively managed care trials involving field cats with either confirmed or strongly suggested diagnoses of FIP demonstrated that oral administration of the nucleoside analog GS-441524, an active component of the multicomponent drug Mutian® Xraphconn MT0901 (PATENT US10988503B1), significantly reduced viral RNA loads in blood, effusion, and feces soon after therapy initiation. Notably, by day 14, no viremic cats had been observed. These results underscore the high efficacy of the treatment. Furthermore, Ab titers consistently decreased throughout therapy [68]. GS-441524, a 1′-cyano-substituted adenine C-nucleoside ribose analog, effectively suppresses viral RNA synthesis. Importantly, GS-441524 and a previously identified 3C-like antiviral protease inhibitor have proven effective against FIPV in both trial settings and cases of spontaneously acquired FIP. However, treating the ocular and CNS manifestations of FIP poses challenges because of limited drug penetration across the blood–brain and blood-eye barriers. Elevated relapse rates in CNS-related FIP cases have been observed with protease inhibitor-based therapies, whereas GS-441524 shows promise in treating ophthalmic and neurological manifestations of FIP. The initial field study using GS-441524 for naturally acquired, non-neurological FIP used a dose of 2 mg/kg, which was found to be insufficient for cats with neurological symptoms [46]. GS-441524, the active metabolite of remdesivir (RDV), acts as an RNA chain terminator for viral RNA-dependent RNA polymerase and strongly inhibits FIPV in both tissue culture and experimental cats. *In vitro* experiments and cases of spontaneous FIP have also revealed that GS-441524 inhibited FIPV replication in cultured Crandell–Rees feline kidney (CRFK) cells and naturally infected feline peritoneal macrophages at a concentration of 1.0 μM while remaining non-toxic to CRFK cells at concentrations up to 100 μM. In an *in vivo* study, cats infected with FIPV (FIPV strain m3c-2 serotype I) received a daily subcutaneous injection of GS-441524 at 5.0 or 2.0 mg/kg BW for 2 weeks [71].

## Infection and Immunity

FCoVs infect both domestic and wild felids globally [72]. Most FECV infections are innocuous and remain unnoticed, resulting in moderate diarrhea [4]. Convincing evidence of FECV-induced chronic infections was first reported in the late 1990s. In this study, naturally ill cats were separated and tested for the virus in their excrement. This study demonstrated that FECV induces persistent asymptomatic infections identical to natural infections [73]. These data demonstrate that FECVs are primarily linked to the digestive system, yet they can also infect monocytes, although not as effectively, and thus disseminate throughout the body [74].

Primary FECV infection is either asymptomatic or associated with transitory diarrhea, which is mild and localized in the lower small and large intestines [75]. FECV levels appear to be low in blood monocytes during the early stages of infection [76]. However, the body’s immunity is not always robust; when the Abs level in the blood decreases, cats become susceptible to reinfection [77]. This secondary infection is similar to the primary infection. Moreover, it is believed that most pathologies observed in FIP are caused by the response of immune cells to viral infections and the immune system’s reaction to the infected cells. In this case, a widespread form of FIP arises because of the failure to establish a T-cell defense against a B-cell reaction. Conversely, cats immunized with this disease may exhibit a strong cellular immune response to mitigate the harmful effects of Abs [16].

An imbalance between T-cell and T-cell immunological responses in B lymphocytes is believed to be one reason that cats cannot fight FIPV infection. Macrophages overexpress CD40, interleukin 6, mRNA, and activate factor B cells, all of which have previously been linked to high Ab reliance. A previous study by Malbon *et al*. [78] has investigated the involvement of T-cell regulatory (Treg) and natural killer (NK) cells in intrinsic and flexible cellular immunity in felines treated with natural FIP. Felines with FIP had significantly fewer Tregs and NK cells in their peripheral blood, mesenteric lymph nodes, and spleens, but their mesentery and kidneys were comparable to those of well-infected and uninfected felines. Healthy cats had more NK cells in their lymph nodes than FIP-fed cats and had decreased toxicity levels. It appears that FIPV infection causes substantial NK cell and Treg depletion and impaired NK cell activity. This may limit the ability of natural defense mechanisms to combat viruses, dampen immunological responses, and cause inflammation [78].

Abs can promote FIPV uptake and multiplication in macrophages, contributing to type 3 hypersensitivity vasculitis (a kind of Ab-mediated immune response) [16]. In the context of FIP, cats exhibiting robust cellular immune responses are considered resistant to the disease, whereas those with predominant humoral immune responses are susceptible. Given FIP’s immune-mediated pathology, what is the rationale for protective immunity against the virus? The converse of protective immunity is non-protective. However, some forms of immunity appear to exist, as there are instances (though rare) of events occurring independently, and seropositivity rates in cats significantly exceed the incidence of clinical disease. It has been established that immune cells in healthy cats exhibit a more robust response to FCoV infection than those in cats with FIP [8].

The Ab-dependent augmentation of viral infection occurs when virus-Ab complexes infect monocytes or macrophages more effectively than viruses alone through receptor-mediated endocytosis. Most healthy cats tested positive for FCOV Abs and never received FIP. Thus, Ab molecules do not cause FIP and are not present, suggesting the presence of FIP [79].

## Animal and Public Health Considerations

Kennedy *et al*. [70] examined FCoV, a positivistic, single-stranded, enveloped coronavirus RNA from the genus *Alphacoronavirus*, which includes infectious (TGEV), canine coronavirus (CCoV), and human coronaviruses (NKT-NL63; HCoV-229E). The COVID-19 viruses causing severe acute respiratory syndrome (SARS), one of the most prevalent diseases, are part of the genus *Betacoronavirus*; however, FCoV does not infect humans. The avirulent and hypervirulent FCoV biotypes also recognized as FIPVs and FECVs, respectively, are included in this group [80]. There is a limited association between SARS-CoV and betacoronavirus infectious agents, which can infect pets and other animals. Nevertheless, multiple domestic and wild animals have tested positive for SARS-CoV-2, indicating potential human-to-animal transmission [81]. Pets such as canines, cats, tigers, lions, and minks have all tested positive for SARS-CoV-2 [81]. This methodology was briefly validated to ensure that the process adapted from human plasma could be replicated in feline serum. Empty cat serum exhibited no analyte retention time signals. The calibration curve (n = 11 non-zero concentrations) demonstrated a linear relationship (r > 0.99) with each concentration maintaining an accuracy of <10% of the nominal value. Antiviral substances were evaluated *in vitro* at doses up to 50 μM. N(4)-hydroxycytidine (NHC) doses above 1.5 μM resulted in notable reductions in well uptake values in ultrastructure of *Felis catus* whole fetus (Fcwf-4) cell, indicating cell toxicity. NHC-associated cytotoxicity has been previously documented. The intravenous RDV dose matched the initial therapeutic dose of 200 mg used to treat patients with COVID-19 [38].

Before receiving molnupiravir (MPV), the cats were fasted, which may have contributed to their nausea. The first human investigation of MPVs revealed that food impacts absorption rates. However, treatment exposure in both fasting and fed states was equivalent to that of other cell lines, with a CC_50_ of approximately 7.5 μM, similar to the utilization in the human cell line. In a manner akin to human observations, MPV was rapidly converted from plasma to NHC, which is the active metabolite. Further, investigation into nausea and ptyalism in two of the three oral RDV groups is necessary. Considerations for adjusting or maintaining doses similar to those used in humans should be explored to mitigate the adverse effects of this medication [82]. Numerous treatments have been proposed for FIP management in cats. Studies indicate that various immunophilins actively engage with antibiotics through the coronavirus NSP1 of cyclophilin. Similar to cyclosporine, a reduction in coronavirus replication has been observed across various genera, including felines, birds, and humans. This underscores the role of the cellular immune stimulant (cyclophilin) in the coronavirus replication process. The authors suggested that non-immunosuppressive cyclosporine derivatives could act as broad-spectrum inhibitors for emerging human coronaviruses and other viral diseases. Coronaviruses are prevalent among humans and livestock worldwide. This drug has a significant inhibitory effect on virus replication [83].

Pentoxifylline is extensively used in FIP because it effectively reduces vasculitis in humans, a crucial aspect of the etiology of FIP. However, a trial involving 23 cats with confirmed FIP showed that pentoxifylline had no impact on life duration, quality of life, or other FIP-related factors in clinical or laboratory settings [69]. Coronaviruses have large genomes with numerous potential target genes, highlighting the need for safe and effective antiviral medications. It is hoped that ongoing research efforts to combat highly lethal coronavirus infections in humans will lead to the development of such drugs [84]. However, since the advent of SARS and near-East MERS, or Middle East respiratory syndrome, interest in coronaviruses as contagious agents has surged. There are similarities between what is known about coronaviruses in animals and emerging, potentially fatal viruses in humans. They continually evolve into new hosts, readily combining with closely related species to create novel viruses and can also change cell tropism and pathogenicity within the same host. This phenomenon, known as Ab-dependent enhancement, poses challenges for vaccines used or tested against various viruses, including dengue virus, feline immunodeficiency virus, and HIV-1 [85].

This difference begins with COVID-19’s attachment to ACE2, an ACE receptor, in humans. The prediction of ACE2 receptor homology between cats and humans, as well as the discovery of identical feline and canine ACE2 receptors, indicates that SARS-CoV infections may also occur in these pets. FCoVs bind to receptors other than the ACE2 receptor observed in felines. FCoV type II uses receptor APN within cells [86]. Cats were shown to be particularly vulnerable to SARS in an experimental study that evaluated the sensitivity of various animal species to the virus [87]. The mechanism underlying type I FCoV infection remains unclear. Lectin on the cell membrane (fDC-SIGN) appears to play a role in the entry of both FCoV types [88]. According to Decaro *et al*. [77], each of these CoVs originates in the human-animal interface, driven by increasing deforestation, jungle encroachment, anthropomorphization of habitats, and consumption of threatened and endangered wildlife [77]. However, the risk of animal-to-human transmission remains unknown [89].

## Conclusion

FIP is a severe and often fatal cat disease caused by FCoV mutations. While FCoV primarily exists as the less virulent FECV, the more pathogenic FIPV represents a distinct biotype with genetic similarities to other viruses, including retroviruses. Only a minority of cats infected with FCoV develop FIP, but the disease is challenging to diagnose due to its nonspecific clinical signs. Ongoing research into its pathogenesis, causative agents, epidemiology, and immunity is essential for developing effective prevention and treatment strategies. FIP affects domestic and wild felids, particularly those under two years old, and presents in two main forms: effusive (wet) and non-effusive (dry).

Although traditionally viewed as untreatable, advancements in therapies, such as GS-441524, have shown promise in reducing viral loads and improving outcomes for FIP patients. Other treatment options, including immunomodulators and antivirals, are also being investigated, though their effectiveness can vary. More research is necessary to identify optimal treatment strategies, especially for cases with neurological or ophthalmic involvement.

FCoV infections can be asymptomatic or cause mild diarrhea, particularly in domestic cats. A robust immune response typically helps prevent FIP, whereas a weakened immune system can increase susceptibility to secondary infections. Studies suggest the interplay between antibodies and the virus may enhance infection risk.

## Authors’ Contributions

TIS, QADA, RAD, DAFS, GNR, VAH, and GPS: Conceptualized the study, collected the literature, and drafted and edited the manuscript. All authors have read and approved the final manuscript.

## References

[1] Thayer V, Gogolski S, Felten S, Hartmann K, Kennedy M, Olah G.A (2022). 2022 AAFP/EveryCat Feline infectious peritonitis diagnosis guidelines. J. Feline Med. Surg.

[2] Si F, Yu R, Dong S, Chen B, Li C, Song S (2024). Towards a safer future:Enhancing vaccine development to combat animal coronaviruses. Vaccines (Basel).

[3] Olsen C.W (1993). A review of feline infectious peritonitis virus:Molecular biology, immunopathogenesis, clinical aspects, and vaccination. Vet. Microbiol.

[4] Tekes G, Thiel H.J (2016). Feline coronaviruses:Pathogenesis of feline infectious peritonitis. Adv. Virus Res.

[5] Xia H, Li X, Zhao W, Jia S, Zhang X, Irwin D.M, Zhang S (2020). Adaptive evolution of feline coronavirus genes based on selection analysis. Biomed. Res. Int.

[6] Poland A.M, Vennema H, Foley J.E, Pedersen N.C (1996). Two related strains of feline infectious peritonitis virus isolated from immunocompromised cats infected with a feline enteric coronavirus. J. Clin. Microbiol.

[7] Tasker S, Addie D.D, Egberink H, Hofmann-Lehmann R, Hosie M.J, Truyen U, Belák S, Boucraut-Baralon C, Frymus T, Lloret A, Marsilio F, Pennisi M.G, Thiry E, Möstl K, Hartmann K (2023). Feline infectious peritonitis:European Advisory Board on Cat Diseases Guidelines. Viruses.

[8] Pedersen N.C (2014). An update on feline infectious peritonitis:Diagnostics and therapeutics. Vet. J.

[9] Lorusso E, Mari V, Losurdo M, Lanave G, Trotta A, Dowgier G, Colaianni M.L, Zatelli A, Elia G, Buonavoglia D, Decaro N (2019). Discrepancies between feline coronavirus antibody and nucleic acid detection in effusions of cats with suspected feline infectious peritonitis. Res. Vet. Sci.

[10] Vennema H, Poland A, Foley J, Pedersen N.C (1998). Feline infectious peritonitis viruses arise by mutation from endemic feline enteric coronaviruses. Virology.

[11] Ouyang H, Liu J, Yin Y, Cao S, Yan R, Ren Y, Zhou D, Li Q, Li J, Liao X, Ji W, Du B, Si Y, Hu C (2022). Epidemiology and comparative analyses of the S Gene on feline coronavirus in central China. Pathogens.

[12] Pedersen N.C, Johnson L, Theilen G.H (1984). Biological behavior of tumors and associated retroviremia in cats inoculated with Snyder-Theilen fibrosarcoma virus and the phenomenon of tumor recurrence after primary regression. Infect. Immun.

[13] Kennedy M, Boedeker N, Gibbs P, Kania S (2001). Deletions in the 7a ORF of feline coronavirus associated with an epidemic of feline infectious peritonitis. Vet. Microbiol.

[14] Lin C.N, Su B.L, Huang H.P, Lee J.J, Hsieh M.W, Chueh L.L (2009). Field strain feline coronaviruses with small deletions in ORF7b associated with both enteric infection and feline infectious peritonitis. J. Feline Med. Surg.

[15] Takano Y, Yamada S, Doki T, Hohdatsu T (2019). Pathogenesis of oral type I feline infectious peritonitis virus (FIPV) infection:Antibody-dependent enhancement infection of cats with type I FIPV via the oral route. J. Vet. Med. Sci.

[16] Pedersen N.C (2009). A review of feline infectious peritonitis virus infection:1963–2008. J. Feline Med. Surg.

[17] Chappuis G, Duret C (1978). Feline infectious peritonitis:Present knowledge. Comp. Immunol. Microbiol. Infect. Dis.

[18] Moyadee W, Sunpongsri S, Choowongkomon K, Roytrakul S, Rattanasrisomporn A, Tansakul N, Rattanasrisomporn J (2024). Feline infectious peritonitis:A comprehensive evaluation of clinical manifestations, laboratory diagnosis, and therapeutic approaches. J. Adv. Vet. Anim. Res.

[19] Martínez J, Reinacher M, Perpiñán D, Ramis A (2008). Identification of group 1 coronavirus antigen in multisystemic granulomatous lesions in ferrets (*Mustela putorius furo*). J. Comp. Pathol.

[20] Boghian V (2023). Morphoclinical and paraclinical features of feline infectious peritonitis (FIP). J. Appl. Life Sci.

[21] Yin Y, Li T, Wang C, Liu X, Ouyang H, Ji W, Liu J, Liao X, Li J, Hu C (2021). A retrospective study of clinical and laboratory features and treatment on cats highly suspected of feline infectious peritonitis in Wuhan, China. Sci. Rep.

[22] Paltrinieri S, Giordano A, Stranieri A, Lauzi S (2021). Feline infectious peritonitis (FIP) and coronavirus disease 19 (COVID?19):Are they similar?. Transbound. Emerg. Dis.

[23] Crawford A.H, Stoll A.L, Sanchez-Masian D, Shea A, Michaels J, Fraser A.R, Beltran E (2017). Clinicopathologic features and magnetic resonance imaging findings in 24 cats with histopathologically confirmed neurologic feline infectious peritonitis. J. Vet. Intern. Med.

[24] Kornegay J.N (19patho8) Feline infectious peritonitis:The central nervous system form. J. Am. Anim. Hosp. Assoc.

[25] Kipar A, Meli M.L (2014). Feline infectious peritonitis:Still an enigma?. Vet. Pathol.

[26] Giori L, Giordano A, Giudice C, Grieco V, Paltrinieri S (2011). Performances of different diagnostic tests for feline infectious peritonitis in challenging clinical cases. J. Small Anim. Pract.

[27] Sweet A.N, André N.M, Stout A.E, Licitra B.N, Whittaker G.R (2022). Clinical and molecular relationships between COVID-19 and feline infectious peritonitis (FIP). Viruses.

[28] Slaviero M, Cony F.G, da Silva R.C, De Lorenzo C, de Almeida B.A, Bertolini M, Driemeier D, Pavarini S.P, Sonne L (2024). Pathological findings and patterns of feline infectious peritonitis in the respiratory tract of cats. J. Comp. Pathol.

[29] Andrew S. E (2000). Feline infectious peritonitis. Vet. Clin. North Am. Small Anim. Pract.

[30] Berg A.L, Ekman K, Belák S, Berg M (2005). Cellular composition and interferon-g expression of the local inflammatory response in feline infectious peritonitis (FIP). Vet. Microbiol.

[31] Evermann J.F, McKeirnan A.J, Ott R.L (1991). Perspectives on the epizootiology of feline enteric coronavirus and the pathogenesis of feline infectious peritonitis. Vet. Microbiol.

[32] Anwer A.Z, Mousa M.R, Halium M.A, Abouelela Y.S, Elsaid H.M (2022). Clinical and pathological studies on feline infectious peritonitis in Egypt. Int. J. Vet. Sci.

[33] Yuya N, Hideo M, Takehisa S, Takashi U, Satoru M, Tsuyosi O (2013). Dysuria Similar to Urethral Atresia in central nervous system-type of feline infectious peritonitis/Type 1 Feline Coronaviruses. J Stage.

[34] Hartmann K (2005). Feline infectious peritonitis. Vet. Clin. Small Anim. Pract.

[35] Brown M.A, Troyer J.L, Pecon-Slattery J, Roelke M.E, O'Brien S.J (2009). Genetics and pathogenesis of feline infectious peritonitis virus. Emerg. Infect. Dis.

[36] De Groot-Mijnes J.D.F, van Dun J.M, van der Most R.G, de Groot R.J (2005). Natural history of a recurrent feline coronavirus infection and the role of cellular immunity in survival and disease. J. Virol.

[37] Chang H.W, Egberink H.F, Halpin R, Spiro D.J, Rottier P.J.M (2012). Spike protein fusion peptide and feline coronavirus virulence. Emerg. Infect. Dis.

[38] Cook S, Wittenburg L, Yan V.C, Theil J.H, Castillo D, Reagan K.L, Williams S, Pham C.D, Li C, Muller F.L, Murphy B.G (2022). An optimized bioassay for screening combined anticoronaviral compounds for efficacy against feline infectious peritonitis virus with pharmacokinetic analyses of GS-441524, remdesivir, and molnupiravir in cats. Viruses.

[39] Takano T, Wakayama Y, Doki T (2019). Endocytic pathway of feline coronavirus for cell entry:Differences in serotype-dependent viral entry pathway. Pathogens.

[40] Gao Y.Y, Wang Q, Liang X.Y, Zhang S, Bao D, Zhao H, Li S.B, Wang K, Hu G.X, Gao F.S (2023). An updated review of feline coronavirus:Mind the two biotypes. Virus Res.

[41] Moyadee W, Jaroensong T, Roytrakul S, Boonkaewwan C, Rattanasrisomporn J (2019). Characteristic clinical signs and blood parameters in cats with feline infectious peritonitis. Agric. Nat. Resour.

[42] Felten S, Hartmann K (2019). Diagnosis of feline infectious peritonitis:A review of the current literature. Viruses.

[43] Rissi D.R (2018). A retrospective study of the neuropathology and diagnosis of naturally occurring feline infectious peritonitis. J. Vet. Diagn. Invest.

[44] Guan X, Li H, Han M, Jia S, Feng B, Gao X, Wang Z, Jiang Y, Cui W, Wang L, Xu Y (2020). Epidemiological investigation of feline infectious peritonitis in cats living in Harbin, Northeast China from 2017 to 2019 using a combination of an EvaGreen-based real-time RT-PCR and serum chemistry assays. Mol. Cell. Probes.

[45] Martinez M.L, Weiss R.C (1993). Detection of feline infectious peritonitis virus infection in cell cultures and peripheral blood mononuclear leukocytes of experimentally infected cats using a biotinylated cDNA probe. Vet. Microbiol.

[46] Dickinson P.J, Bannasch M, Thomasy S.M, Murthy V.D, Vernau K.M, Liepnieks M, Montgomery E, Knickelbein K.E, Murphy B, Pedersen N.C (2020). Antiviral treatment using the adenosine nucleoside analogue GS ?441524 in cats with clinically diagnosed neurological feline infectious peritonitis. J. Vet. Intern. Med.

[47] Djani D.M, Thomas W.B (2019). What is your neurologic diagnosis?. J. Am. Vet. Med. Assoc.

[48] Müller T.R, Penninck D.G, Webster C.R.L, Conrado F.O (2023). Abdominal ultrasonographic findings of cats with feline infectious peritonitis:An update. J. Feline Med. Surg.

[49] Aydin H, Yildirim S (2019). Investigation of the relation between feline infectious peritonitis and retroviruses in cats. GSC Biol. Pharm. Sci.

[50] Schmidt M, Ondreka N (2019). Hydrocephalus in animals. Pediatric Hydrocephalus.

[51] Park S, Bae Y, Choi J (2015). Pleuropneumonia in a cat with feline infectious peritonitis. J. Vet. Clin.

[52] Dunbar D, Kwok W, Graham E, Armitage A, Irvine R, Johnston P, McDonald M, Montgomery D, Nicolson L, Robertson E, Weir W, Addie D.D (2019). Diagnosis of non-effusive feline infectious peritonitis by reverse transcriptase quantitative PCR from mesenteric lymph node fine-needle aspirates. J. Feline Med. Surg.

[53] Doenges S.J, Weber K, Dorsch R, Fux R, Fischer A, Matiasek L.A, Matiasek K, Hartmann K (2016). Detection of feline coronavirus in cerebrospinal fluid for diagnosis of feline infectious peritonitis in cats with and without neurological signs. J. Feline Med. Surg.

[54] Li X, Scott F.W (1994). Detection of feline coronaviruses in cell cultures and in fresh and fixed feline tissues using polymerase chain reaction. Vet. Microbiol.

[55] Haake C, Cook S, Pusterla N, Murphy B (2020). Coronavirus infections in companion animals:Virology, epidemiology, clinical and pathologic features. Viruses.

[56] Sevinç M, Ok M, Baş T.M (2020). Coronavirus infection in cats, Eurasian Journal of Veterinary Sciences.

[57] Murphy B.G, Castillo D, Neely N.E, Kol A, Brostoff T, Grant C.K, Reagan K.L (2024). Serologic, virologic and pathologic features of cats with naturally occurring feline infectious peritonitis enrolled in antiviral clinical trials. Viruses.

[58] Hayashi T, Utsumi F, Takahashi R, Fujiwara K (1980). Pathology of non-effusive type feline infectious peritonitis and experimental transmission. Nihon Juigaku Zasshi.

[59] Tasker S (2018). Diagnosis of feline infectious peritonitis:Update on evidence supporting available tests. J. Feline Med. Surg.

[60] Hohdatsu T, Okada S, Koyama H (1991). Characterization of monoclonal antibodies against feline infectious peritonitis virus type II and antigenic relationship between feline, porcine, and canine coronaviruses. Arch. Virol.

[61] Stoddart M.E, Gaskell R.M, Harbour D.A, Gaskell C.J (1988). Virus shedding and immune responses in cats inoculated with cell culture-adapted feline infectious peritonitis virus. Vet. Microbiol.

[62] Pedersen N.C (1987). Virologic and immunologic aspects of feline infectious peritonitis virus infection. Adv. Exp. Med. Biol.

[63] Berliner E.A (2021). Feline coronavirus and feline infectious peritonitis. Infectious Disease Management in Animal Shelters.

[64] Delaplace M, Huet H, Gambino A, Le Poder S (2021). Feline coronavirus antivirals:A review. Pathogens.

[65] Barker E.N, Tasker S (2020). Advances in molecular diagnostics and treatment of feline infectious peritonitis. Adv. Small Anim. Care.

[66] Berry M.L (2001). Feline infectious peritonitis. Feline Internal Medicine Secrets.

[67] Meli M.L, Spiri A.M, Zwicklbauer K, Krentz D, Felten S, Bergmann M, Dorsch R, Matiasek K, Alberer M, Kolberg L, von Both U, Hartmann K, Hofmann-Lehmann R (2022). Fecal feline coronavirus RNA shedding and spike gene mutations in cats with feline infectious peritonitis treated with GS-441524. Viruses.

[68] Khoo C.K, Dahlan R, Mat Desa Z.M, Syarina P.N.A, Salim S.S.H, Barker Z, Abu Hassan M.H.A, Hassan R, Mohd Saeid F.H.M (2022). Molecular detection of lumpy skin disease virus in Malaysia 2021. Int. J. Infect. Dis.

[69] Fischer Y, Ritz S, Weber K, Sauter?Louis C, Hartmann K (2011). Randomized, placebo controlled study of the effect of propentofylline on survival time and quality of life of cats with feline infectious peritonitis. J. Vet. Intern. Med.

[70] Kennedy M.A (2020). Feline infectious peritonitis:Update on pathogenesis, diagnostics, and treatment. Vet. Clin. North Am. Small Anim. Pract.

[71] Izes A.M, Yu J, Norris J.M, Govendir M (2020). Current status on treatment options for feline infectious peritonitis and SARS-CoV-2 positive cats. Vet. Q.

[72] Wasieri J, Schmiedeknecht G, Förster C, König M, Reinacher M (2009). Parvovirus infection in a Eurasian lynx (*Lynx lynx*) and in a European wildcat (*Felis silvestris silvestris*). J. Comp. Pathol.

[73] Tang Y.W, Procop G.W, Persing D.H (1997). Molecular diagnostics of infectious diseases. Clin. Chem.

[74] Lewis C.S, Porter E, Matthews D, Kipar A, Tasker S, Helps C.R, Siddell S.G (2015). Genotyping coronaviruses associated with feline infectious peritonitis. J. Gen. Virol.

[75] Vogel L, Van der Lubben M, Te Lintelo E.G, Bekker C.P.J, Geerts T, Schuijff L.S, Grinwis G.C.M, Egberink H.F, Rottier P.J.M (2010). Pathogenic characteristics of persistent feline enteric coronavirus infection in cats. Vet. Res.

[76] Kipar A, Meli M.L, Baptiste K.E, Bowker L.J, Lutz H (2010). Sites of feline coronavirus persistence in healthy cats. J. Gen. Virol.

[77] Decaro N, Martella V, Saif L.J, Buonavoglia C (2020). COVID-19 from veterinary medicine and one health perspectives:What animal coronaviruses have taught us. Res. Vet. Sci.

[78] Malbon A.J, Russo G, Burgener C, Barker E.N, Meli M.L, Tasker S, Kipar A (2020). The effect of natural feline coronavirus infection on the host immune response:A whole-transcriptome analysis of the mesenteric lymph nodes in cats with and without feline infectious peritonitis. Pathogens.

[79] Hartmann K, Binder C, Hirschberger J, Cole D, Reinacher M, Schroo S, Frost J, Egberink H, Lutz H, Hermanns W (2003). Comparison of different tests to diagnose feline infectious peritonitis. J. Vet. Intern. Med.

[80] Addie D, Houe L, Maitland K, Passantino G, Decaro N (2020). Effect of cat litters on feline coronavirus infection of cell culture and cats. J. Feline Med. Surg.

[81] Halfmann P.J, Hatta M, Chiba S, Maemura T, Fan S, Takeda M, Kinoshita N, Hattori S.I, Sakai-Tagawa Y, Iwatsuki-Horimoto K, Imai M, Kawaoka Y (2020). Transmission of SARS-CoV-2 in domestic cats. N. Engl. J. Med.

[82] Beigel J.H, Tomashek K.M, Dodd L.E, Mehta A.K, Zingman B.S, Kalil A.C, Hohmann E, Chu H.Y, Luetkemeyer A, Kline S, Lopez de Castilla D, Finberg R.W, Dierberg K, Tapson V, Hsieh L, Patterson T.F, Paredes R, Sweeney D.A, Short W.R, Touloumi G, Lye D.C, Ohmagari N, Oh M.-D, Ruiz-Palacios G.M, Benfield T, Fätkenheuer G, Kortepeter M.G, Atmar R.L, Creech C.B, Lundgren J, Babiker A.G, Pett S, Neaton J.D, Burgess T.H, Bonnett T, Green M, Makowski M, Osinusi A, Nayak S, Lane H.C, ACTT-1 Study Group Members (2020). Remdesivir for the treatment of Covid-19 - final report. N. Engl. J. Med.

[83] Pfefferle S, Schöpf J, Kögl M, Friedel C.C, Müller M.A, Carbajo-Lozoya J, Stellberger T, von Dall'Armi E, Herzog P, Kallies S, Niemeyer D, Ditt V, Kuri T, Züst R, Pumpor K, Hilgenfeld R, Schwarz F, Zimmer R, Steffen I, Weber F, Thiel V, Herrler G, Thiel H.J, Schwegmann-Wessels C, Pöhlmann S, Haas J, Drosten C, von Brunn A (2011). The SARS-coronavirus-host interactome:Identification of cyclophilins as target for pan-coronavirus inhibitors. PLOS Pathog.

[84] Zhang X, Chu H, Wen L, Shuai H, Yang D, Wang Y, Hou Y, Zhu Z, Yuan S, Yin F, Chan J.F.W, Yuen K.Y (2020). Competing endogenous RNA network profiling reveals novel host dependency factors required for MERS-CoV propagation. Emerg. Microbes Infect.

[85] Sharun K, Sircar S, Malik Y.S, Singh R.K, Dhama K (2020). How close is SARS-CoV-2 to canine and feline coronaviruses?. J. Small Anim. Pract.

[86] Guo H, Guo A, Wang C, Yan B, Lu H, Chen H (2008). Expression of feline angiotensin converting enzyme 2 and its interaction with SARS-CoV S1 protein. Res. Vet. Sci.

[87] Shi J, Wen Z, Zhong G, Yang H, Wang C, Huang B, Liu R, He X, Shuai L, Sun Z, Zhao Y, Liu P, Liang L, Cui P, Wang J, Zhang X, Guan Y, Tan W, Wu G, Chen H, Bu Z, Bu Z (2020). Susceptibility of ferrets, cats, dogs, and other domesticated animals to SARS-coronavirus 2. Science.

[88] Van Hamme E, Desmarets L, Dewerchin H.L, Nauwynck H.J (2011). Intriguing interplay between feline infectious peritonitis virus and its receptors during entry in primary feline monocytes. Virus Res.

[89] Sit T.H.C, Brackman C.J, Ip S.M, Tam K.W.S, Law P.Y.T, To E.M.W, Yu V.Y.T, Sims L.D, Tsang D.N.C, Chu D.K.W, Perera R.A.P.M, Poon L.L.M, Peiris M (2020). Infection of dogs with SARS-CoV-2. Nature.

